# Functionalized CNTs with DOPO and Silicon Containing Agents: Effective Reinforcer for Thermal and Flame Retardant Properties of Polystyrene Nanocomposites

**DOI:** 10.3389/fchem.2020.627642

**Published:** 2021-02-10

**Authors:** Congling Shi, Xiaodong Qian, Jingyun Jing, Honglei Che

**Affiliations:** Beijing Key Lab of MFPTS, China Academy of Safety Science and Technology, Beijing, China

**Keywords:** nanocomposites, polystyrene, flame retardants, CNTs, DOPO

## Abstract

DOPO and silicon containing agents modified multiwalled carbon nanotubes (MCNTs) were synthesized through sol-gel process and MCNTs are introduced into polystyrene (PS) through *in situ* polymerization. TEM observations and FTIR results of MCNTs demonstrated that the MCNT nanofillers were coated with the organic/inorganic flame retardant compound. Moreover, the TEM results of the composites indicate that MCNTs dispersed in polystyrene PS matrix uniformly due to the modification. The PS/MCNTs composites showed improved thermal stability as well as flame retardant properties in comparison with PS/CNTs composites, which are due to the good dispersion of MCNT in the PS matrix. MCNTs in the PS matrix can also reduce the peak heat release rate, total heat release and improve the smoke suppression performance. The improved flame retardant properties are attributed to the char reinforcing effect of CNTs, which can provide enough time for MCNTs and organic/inorganic compound to trap the degradation of polymer chains and catalyze the formation of char. The char layers can not only serve as an efficient insulating barrier to reduce the exposure of PS matrix to heat source but also retard the releasing of combustible gas.

## Introduction

Since 1991, carbon nanotubes (CNTs) have became a major interest of research all over the world. The properties of CNTs include the resistance to acid, high adsorption capacity and possibility to control surface chemistry. Those special properties make CNTs applicable to a wide range of potential applications, taking electronics, polymer nanocomposites and medical devices for example (Esawi et al., 2007; Gao et al., 2009; Zhang et al., 2018; Ali et al., 2019). Among various polymer nanocomposites, taking the advantage of the flame retardant effect of CNTs is one of the most promising research directions (Hapuarachchi et al., 2010; Ji et al., 2018). Kashiwagi found that CNTs can reduce the heat release rate of polypropylene at very low CNTs loading, and the network structured layers of CNTs can act as excellent thermal insulation char layers to protect the underlying polypropylene from external heat and radiation (Kashiwagi et al., 2005). However, the agglomeration of CNTs usually results in the reduced flame retardant and mechanical properties, which are due to strong Vander Waals forces and π–π interactions among CNTs. As a result, one of the major challenges for preparing polymer composites is to disperse the CNTs in the polymer matrices individually.

As for polymer composites based on CNTs, many methods have been developed to improve the dispersion of CNTs in the polymers composites through surface modification. Among those methods, the covalent and noncovalent functionalization are common modification method (Kim et al., 2006; Gupta et al., 2015). Generally, the noncovalent functionalization of CNTs can confer the special properties of CNTs without changing the structural characteristics, but the dispersion of CNTs in the polymer composites is usually not very ideal (Huang et al., 2019a; Huang et al., 2019b). As for the covalent functionalization, the solubility and compatibility of CNTs in the polymer matrix can be improved significantly, and various functional organic groups can be adopted to modified CNTs, resulting in the uniform dispersion of CNTs in the polymer composites.

The organic/inorganic hybrids materials based on silicon are generally prepared through the sol-gel reaction. As for the organosilane, the organic groups can be varied according their application. As for the flame retardancy, the organic groups of organosilane can be changed to phosphorus based groups (SforA et al., 1999; Kurayama et al., 2010). Among the phosphorus based flame retardants, 9,10-dihydro-9-oxa-10-phosphaphenanthrene-10-oxide (DOPO) and its derivative have attracted great attention in the field of flame retardant due to their high flame retardant efficiency to polymer materials and high thermal stability (Perret et al., 2011; Salmeia. et al., 2015). In the previous work, novel organic/inorganic flame retardants based on silicane and DOPO was designed, and the flame retardants play their flame retardant roles in not only condensed phase but also gases phase (Qian et al., 2013). Due to the existence of silane structure, the novel organic/inorganic flame retardants can be use as a good modifier for the inorganic fillers.

As a common engineering plastics, polystyrene (PS) has outstanding properties such as low density, excellent mechanical properties, chemical resistance and easy processing molding. Due to those outstanding properties, PS has been broadly used in various fields such as decoration, transportation, etc., (Qian et al., 2000; Jürgen et al., 2009; Shi et al., 2019) However, PS is high flammability, which limits its further application in the areas with high fire safety requirements. In order to improve its flame retardant properties of PS, various flame retardants such as ammonium polyphosphate, aluminum phosphate and decabromodiphenyl ethane have been incorporated into the PS matrix (Wang et al., 2002; Braun et al., 2004; Li et al., 2020). Due to the environmental problems, researchers have been exploring novel halogen-free flame retardants such as novel phosphorus based flame retardants or nano-fillers flame retardants.

In this manuscript, DOPO and silicon containing agents modified MCNTs were prepared through sol-gel process and the MCNTs was incorporated into PS matrix through free radical addition polymerization. The TEM results of the composites indicate that MCNTs distributed within the PS matrix uniformly. Compared with PS/CNTs composites, PS/MCNTs composites showed improved thermal stability and flame retardant properties, which are due to the improved dispersion and flame retardant effect of DOPO and silicon containing agents.

## Experimental Section

### Materials

Vinyl trimethoxysilane was purchased from Shenmao new material Co., (Guangzhou, China). 9,10-Dihydro-9-oxa-10-phosphaphenanthrene-10-oxide (DOPO) was supplied by Shandong Mingshan Fine Chemical Industry Co., Ltd. (Shandong, China). Styrene and azodiisobutyronitrile (AIBN) were purchased from Sinopharm Chemical Reagent Co., Ltd. (Shanghai, China). Moreover, carbon nanotubes (CNTs) were supplied by Chengdu Organic Chemicals Co., Ltd.

### Preparation of Multiwalled Carbon Nanotubes

**SCHEME 1:**
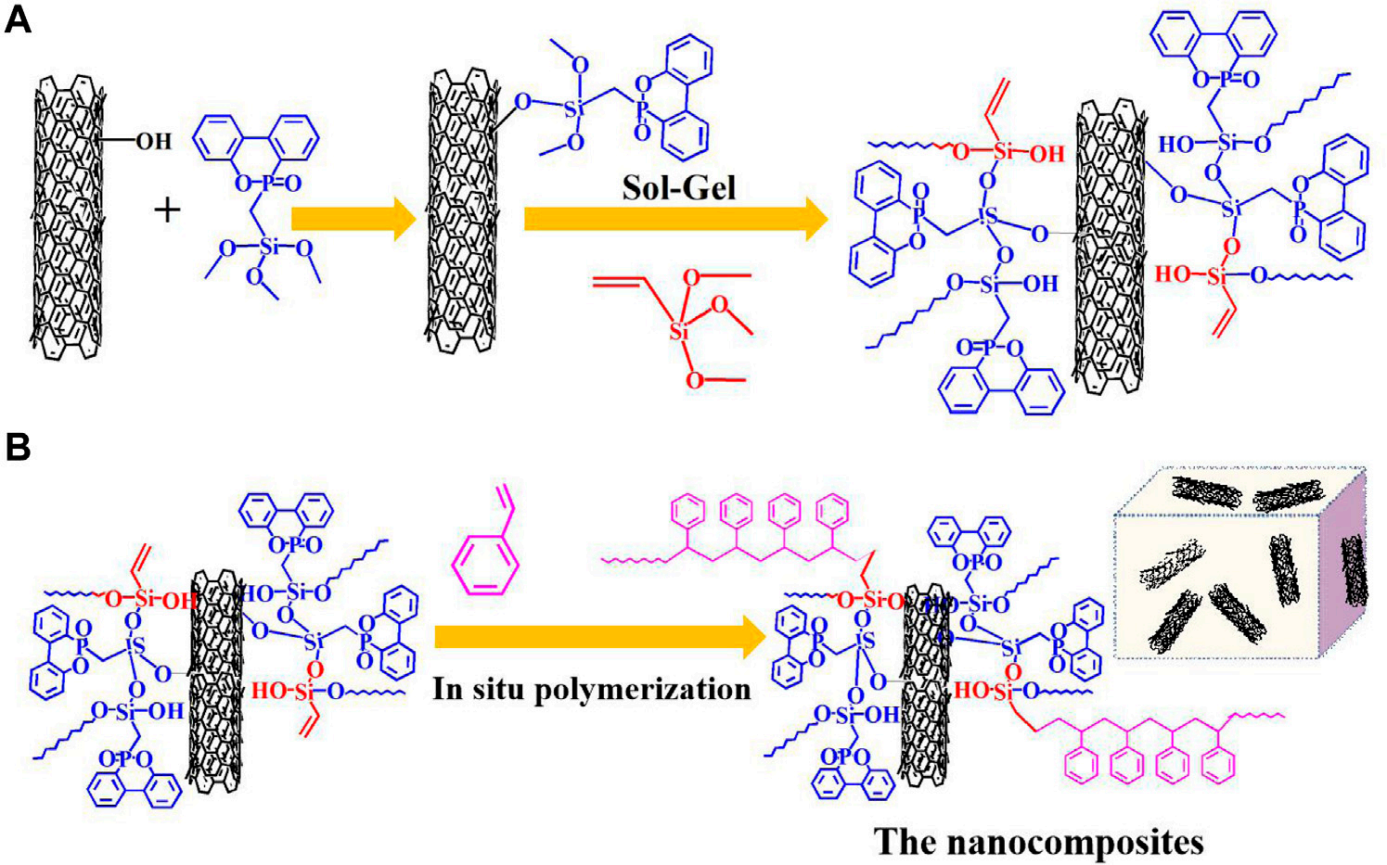
Preparation of MCNTs and PS/MCNTs composites: **(A)** preparation routes of MCNTs; **(B)** preparation routes of PS/MCNTs composites.

The DOPO-VTS was synthesized according to our previous report (Qian et al., 2013). In a 250 ml three-necked flask, CNTs (2 g), DOPO-VTS (5 g) and THF (100 ml) were mixed. After the mixtures were saturated with nitrogen atmosphere in case of mechanical mixing, the temperature of the mixtures was increased to 60°C. After stirring for 12 h, 2 g vinyltrimethoxysilane (A171) was added to the mixtures by drop addition, then the mixtures of ammonia (1 ml) and water (10 ml) were dropped into the three-necked flask. After that, the black powders were obtained by filtration. Moreover, the black powders were purified by washing with THF for several times and dried at 60°C in vacuum for 12 h. The black powders named MCNTs were obtained (2.2 g). Scheme 1A illustrates the synthesis routs of MCNTs.

### Preparation of Flame Retardant Polystyrene Composites

Generally, MCNTs (2.0 g) and styrene monomers (98.0 g) were added into a three-necked flask and kept in ultrasonic state for 120 min. After MCNTs are dispersed well in the mixtures, AIBN (0.2 g) was added and the mixtures were kept stirring at 80°C for 60 min until viscous paste formed. Then, the mixtures were transferred into a mold and kept heating at 60°C for 48 h. Scheme 1B illustrates the synthesis routs of PS/MCNTs composites. Then the PS/MCNTs are obtained and other composites are prepared by the same way according to Table 1.

**TABLE 1:** The compositions, TGA and cone data of the composites.

Samples	Nanofillers (wt.%)	pHRR (W/g)	THR (kJ/g)	T_10%_°C	T_50%_°C	Char residues (%)
PS	0	1,003	120	331	375	0.96
PS/CNTs	2	932	121	350	396	0.97
PS/MCNTs-1	1	896	121	354	402	1.16
PS/MCNTs-2	2	745	111	361	403	3.09

### Characterization

The FTIR spectroscopy was recorded with Nicolet 6700 FT-IR spectrophotometer and the wavelength range of the FTIR spectroscopy was 4000–500 cm^−1^.

The structures of MCNTs and the dispersions of CNTs in the composites were investigated by transmission electron microscopy (TEM) (JEOL JEM-2100 instrument).

The thermal stability of the composites was investigated by TGA Q5000 IR thermal gravimetric analyzer (TA Instruments). About 4∼10 mg of the composites was heated from room temperature to 800°C.

The flame retardant proerties of the polymer composites was studied by cone calorimeter according to ISO 5660. The heat flux is 50 kW·m^−1^ and the dimensions of the samples are 100 × 100 × 3 mm^3^ (Yu et al., 2016; Shi et al., 2019a; Shi et al., 2019b; Shi et al., 2019c; Lin et al., 2020; Yu et al., 2021). The samples are tested three times and the average value is selected.

The char layers were investigated by Raman spectroscopy measurements (SPEX-1403 laser Raman spectrometer) at room temperature.

## Results and Discussion

### Characterizations of Carbon Nanotubes and Multiwalled Carbon Nanotubes

**FIGURE 1:**
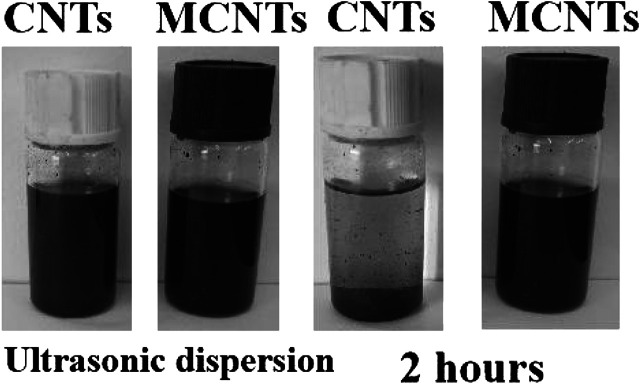
Digital photos of CNTs and MCNTs dispersion in THF at different time.

**FIGURE 2:**
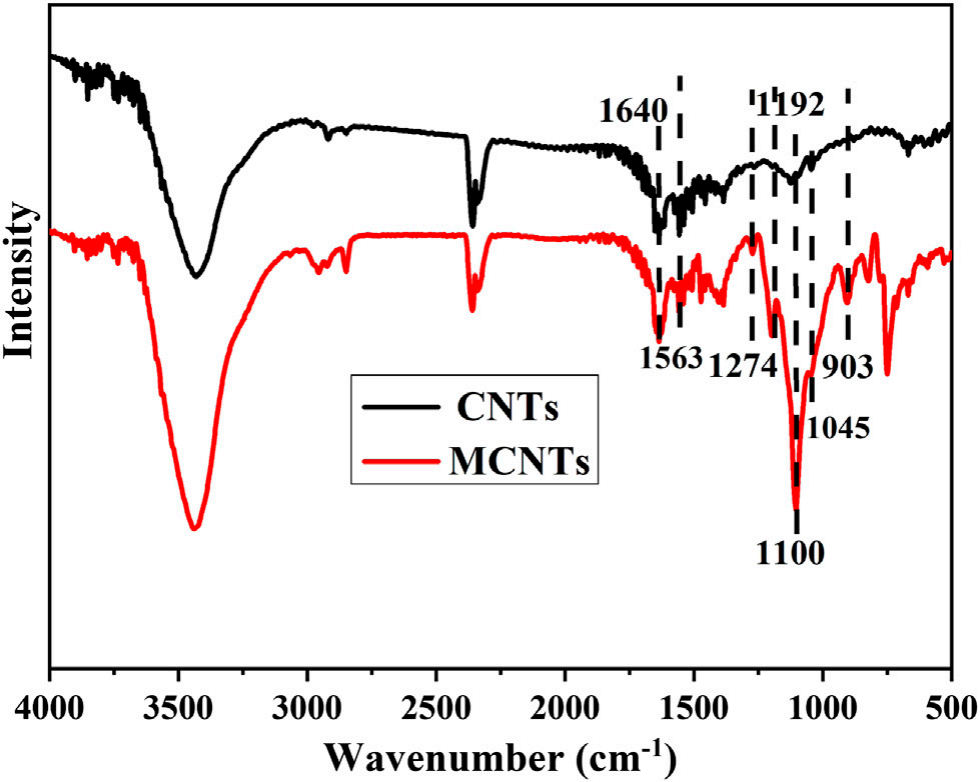
FTIR of CNTs and MCNTs.

The dispersion of CNTs and MCNTs in organic solvent can reflect the surface modification performance, the decentralized states of CNTs and MCNTs in tetrahydrofuran (THF) were investigated at different time. Figure 1 shows the dispersion state of CNTs and MCNTs at a concentration of 1 mg·ml^−1^ after sonication in THF. The DOPO based organic/inorganic compounds on the surface of CNTs have significant influence on the dispersibility of MCNTs in THF. After ultrasonic treatment, the CNTs and MCNTs were well-dispersed in THF, exhibiting the black color. After 2 h, the CNTs precipitated at the bottom while MCNTs were well-dispersed in THF, which are due to the attractive force between the polar organic groups on the surface of MCNTs and organic solution. Figure 2 shows the FTIR spectra of MCNTs and CNTs. As for the MCNTs, the peaks for the stretching vibrations of P-O-Ph appear at both 1,045 cm^−1^ and 903 cm^−1^, the peaks at 1,274 cm^−1^ are due to the stretching vibrations of P=O, and the peak at 1,640 cm^−1^ corresponds to the C=C bonds in the DOPO structures. Moreover, the characteristic peak for P-Ph bonds appeared at 1,563 cm^−1^ and the peaks at around 1,100 cm^−1^ are due to Si-O-C and Si-O-Si bonds (Ciesielski et al., 2008; Wang et al., 2011; Lu et al., 2017). The peaks at 1,560 cm^−1^ are due to the characteristic peak of benzene ring in the DOPO structures, indicating the graft modification of organic/inorganic compound containing DOPO structures and C=C bonds onto the surface of MCNTs.

### Morphology of Carbon Nanotubes, Multiwalled Carbon Nanotubes and the Polystyrene/Multiwalled Carbon Nanotubes Composites

**FIGURE 3:**
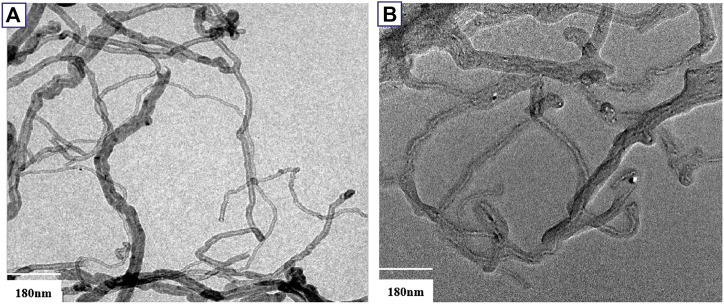
TEM images of CNTs **(A)** and MCNTs **(B)**.

**FIGURE 4:**
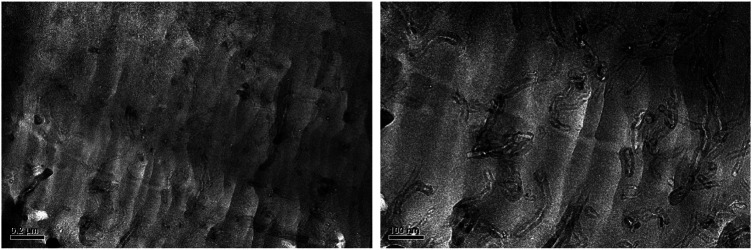
a: TEM images of the PS/MCNTs composites.

The TEM images of CNTs and MCNTs are presented in Figure 3. As for CNTs, it has curved shape. After the modification (Figure 3B), it is found that the diameters of CNTs increased compared with CNTs. The TEM results provide the direct evidence for modification of CNTs with DOPO based flame retardants. The properties of the CNTs based composites depend strongly on the dispersion and interface interaction between CNT and the polymer matrix. Therefore, TEM was adopted to investigate the dispersion state of the MCNTs in the composites, as shown in Figure 4. It’s found that the boundary of MCNTs are obscure and the MCNTs are dispersed well in the PS matrix, which are due to the modification of CNTs with the organic/inorganic flame retardants. Generally, the uniform dispersion characteristics of MCNTs in the PS matrix are due to the good interfacial interaction between the MCNTs and polystyrene molecular chains. The organic/inorganic groups on the surface of CNTs have good interaction with the polystyrene molecular chains due to the formation of covalent bond, resulting in the uniform dispersion of CNTs in the polystyrene matrix.

### Thermo-Oxidation Decomposition Performance of the Composites

**FIGURE 5:**
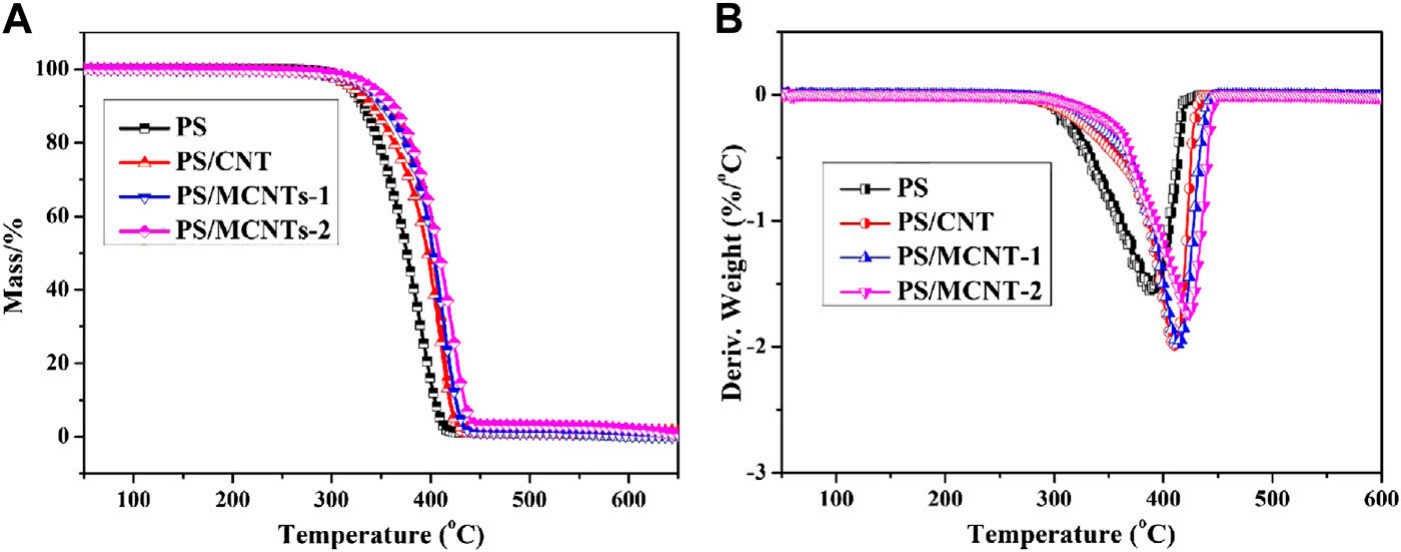
TGA/DTG profiles for PS and its composites as function of temperature under air atmosphere.

The thermo-oxidation properties of PS and its composites were investigated by TGA under air conditions in Figure 5 and the corresponding data are collected in Table 1. The 10% weight loss temperatures are regarded as the onset temperatures (T_10%_) while the 50% weight loss temperature are on behalf of due to half degradation temperatures (T_50%_). The TEM results of PS/MCNTs composites indicate that there are no agglomerates of MCNTs in the PS matrix. Due to the high thermal conductivity of CNTs, the heat usually focus on the domains, resulting in the asymmetric distribution of temperature. As for pure PS, the T10% of PS/MCNTs composites is higher than those of pure PS. Furthermore, it can be observed that there are no residues at 500°C for pure PS, while the corresponding char residues for PS/MCNTs-2 composites at 500°C increase to 3.09wt%. Moreover, the half degradation temperatures (T_50%_) of MCNTs modified PS composites are improved significantly. Only 2.0 wt% MCNTs in the composites result in 28°C increase of T50%. The TGA results indicate that the improved thermo-oxidative stability of the composites against the thermal oxidation are due to the stable char layers formed during the thermal degradation process, attributing to the good dispersion of MCNTs and the organic/inorganic flame retardants on the surface of CNTs (Xing et al., 2016). Generally, the MCNTs can act as high-temperature stabilizers and the organic/inorganic flame retardants can catalyze the formation of stable char layers. Moreover, the physical barrier effect of char layers can provide enough time for MCNTs and DOPO based compounds to trap the degrading polymer radicals and inhibit thermo-oxidative degradation, resulting good flame retardant efficiency.

### The Fire Hazards of the Composites

**FIGURE 6:**
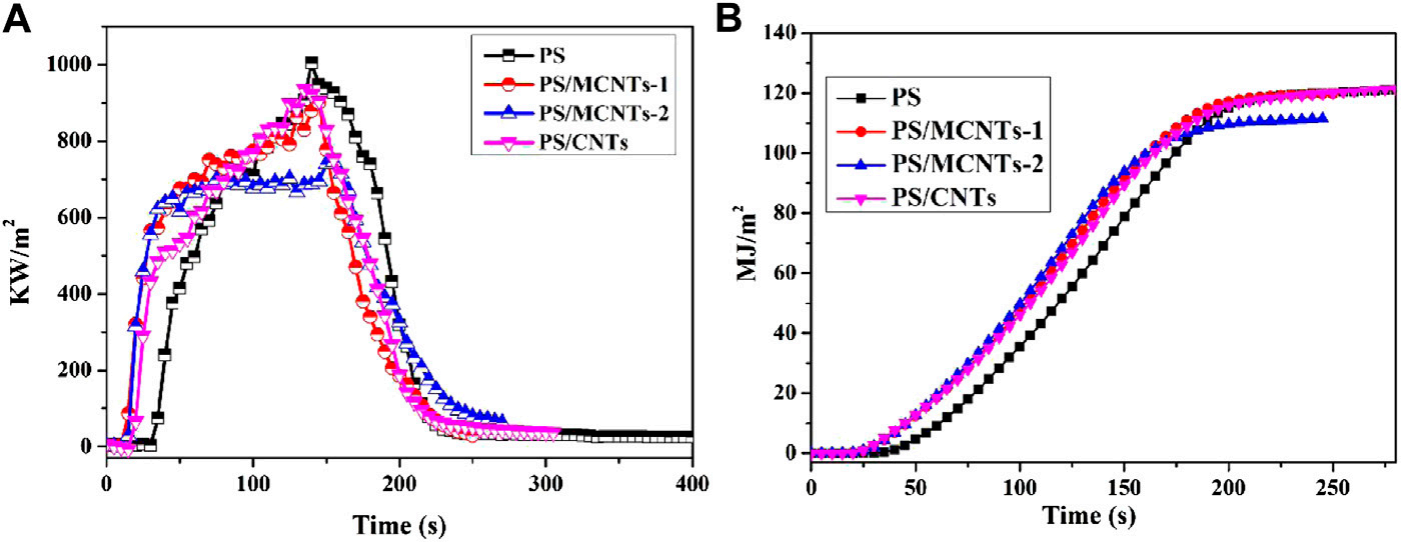
Cone calorimeter test results: HRR and THR curves of PS and its composites.

The fire hazards of materials are investigated by the cone calorimeter, which can show important fire risk parameters such as heat release rate (HRR) and total heat release (THR). The HRR and THR curves of neat PS and its composites at the heat flux of 50 kW/m^2^ are shown in Figure 6, and the corresponding data are summarized in Table 1. As for PS, it has high pHRR (1,003 kW·m^−2^) value, and the HRR peak is at 139.2 s. The incorporation of CNTs or MCNTs into PS resulted in the reduced pHRR value and flatter HRR curves. It can be found that the pHRR of PS/CNTs composites decreases from 1,003 to 929 kW·m^−2^, which is a small decrease. Moreover, the incorporation of CNTs into PS composites has few effects on the reduction of THR. It’s found that the incorporation of MCNTs has better effect on the reduction of pHRR due to the modification of CNTs with the organic/inorganic compound. The THR is further reduced after the 2 wt% of MCNTs was introduced. PS/MCNTs-2 composites have the lowest THR value, which is 7.5% reduction compared with virgin PS. Therefore, the reduced pHRR and THR values of the composites are due to the stable char layers, which can not only prevent the releasing of combustible gas but also the heat release.

### The Investigation of the Char Layers

**FIGURE 7:**
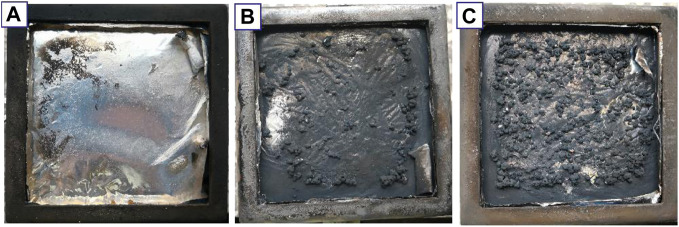
Digital photo of the char residues of PS **(A)**, PS/CNTs **(B)** and PS/MCNTs **(C)**

The improved fire safety properties of PS/CNTs and PS/MCNTs composites are probably due to the good carbonization effect and the char reinforcing effect of CNTs. From the TGA results, it is obvious that MCNTs can improve the char residues and the thermal stability of PS, which is in accordance with the high residual chars after the cone calorimeter test. From Figure 7, it is obvious that PS/MCNTs composites have higher char residues after the cone calorimeter test. The stable char layers with MCNTs are good for CNTs and DOPO based organic/organic flame retardants to trap the degrading polymer radicals and inhibit the further thermal degradation of the composites. Generally, the char layer resulting from the carbonization reaction can act as an insulating barrier to reduce the exposure of PS matrix to an external heat source, resulting in improved flame retardant properties.

**FIGURE 8:**
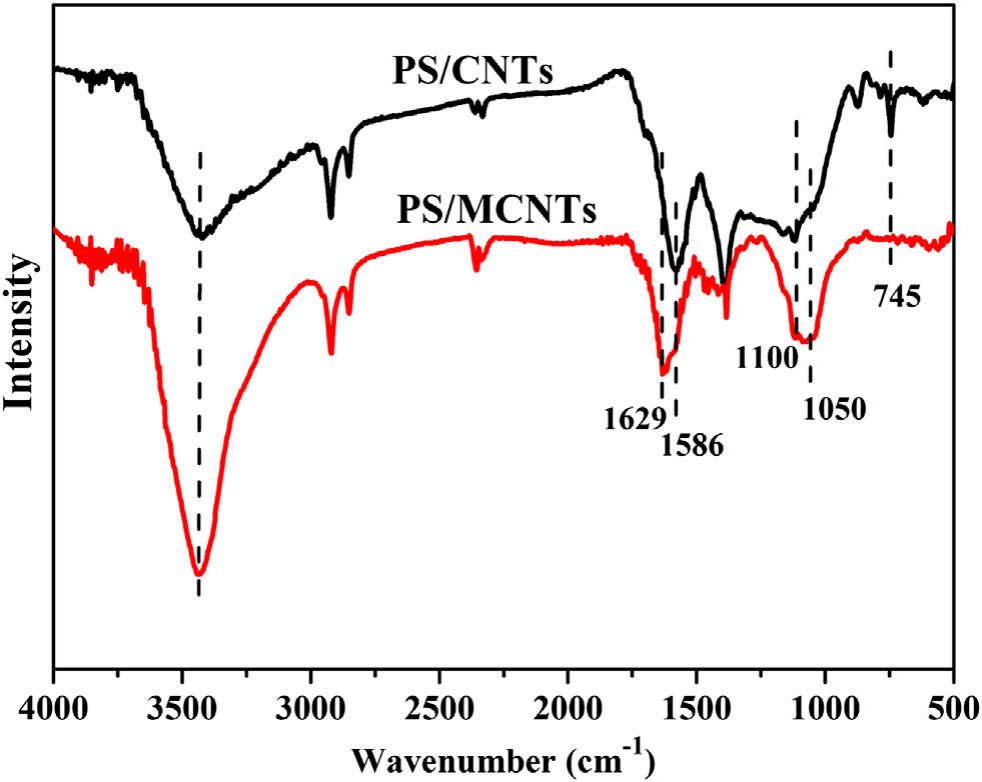
FTIR of the char residues the char residues of PS/CNTs and PS/MCNTs composites.

The FTIR spectra of the char residues of PS and its composites after cone calorimeter test are shown in Figure 8. Compared with the char residues of PS/CNTs composites, the broad peaks of the PS/MCNTs composites at around 1,100 cm^−1^ for the stretching vibration of P=O appears. Moreover, the intensity of the peak at 1,050 cm^−1^ (P-O-P, P-O-C or Si-O-Si bonds) became strength, indicating the formation of the phosphorus-silicon based char layers. Meanwhile, the peak at 1,586 cm^−1^ for the stretching vibrations of C=C in the aromatic compounds of PS/CNTs composites shifts to higher wavenumber, indicating the DOPO based flame retardants influence the structure of char residues (Wu et al., 2009; Qian et al., 2012). Generally, the FTIR results of the char indicate that stable char layers based on the silicon and phosphorus are formed, and the stable char layers can act as an efficient barrier layers to retard the flammable gases from releasing and PS matrix from the exposure of heat.

**FIGURE 9:**
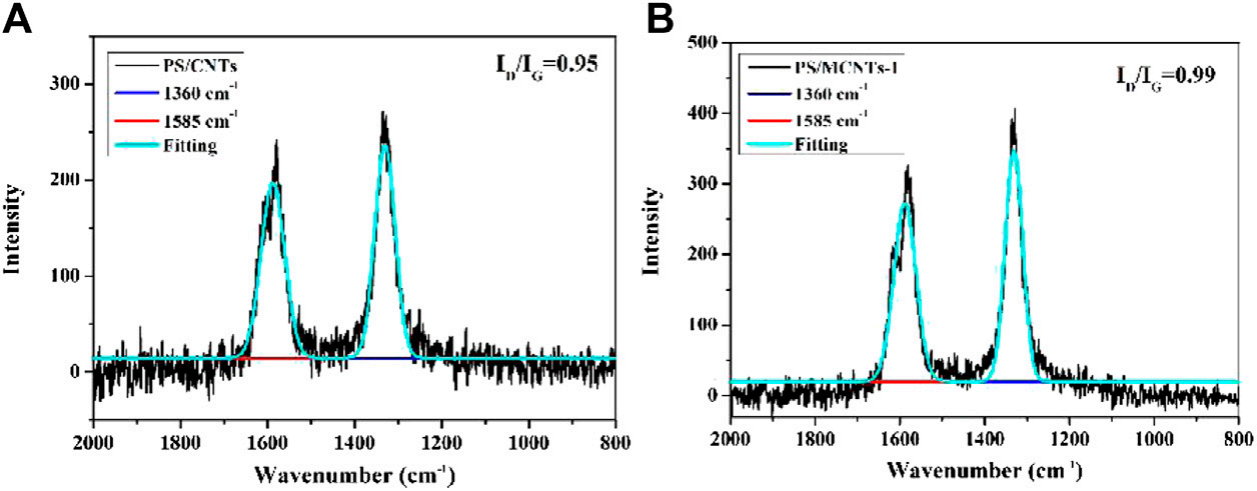
Raman spectra of the char residues of PS/CNTs and PS/MCNTs-1 composites.

**FIGURE 10:**
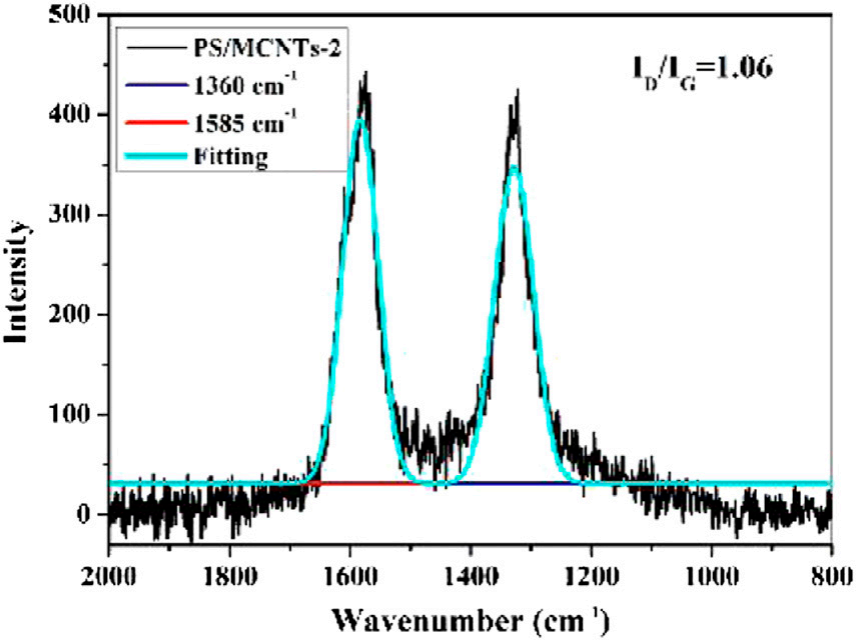
Raman spectra of the char residues of PS/MCNTs-2 composites.

Raman spectroscopy is used to analyze carbonaceous materials, as shown in Figure 9. The Raman spectroscopy of the char layers usually exhibits two strong peaks at about 1,360 and 1,585 cm^−1^. The peak at 1,360 cm^−1^ (the D band) corresponds to disordered graphite or glassy carbon, and the peak at 1,585 cm^−1^ (G band) is due to the aromatic layers of crystalline graphite. Generally, the graphitization degree of the char layers is evaluated by the ratio of the intensity of the D and G bands (I_D_/I_G_), and the lower ratio of I_D_/I_G_ indicate higher graphitization degree of the char layers (Brian et al., 2005; Sadezky et al., 2005; Shi et al., 2020; Zhang, et al., 2020). As shown in Figure 10, the I_D_/I_G_ ratio follows PS/CNTs < PS/MCNTs-1 < PS/MCNTs-2, indicating that the highest graphitization degree is PS. However, the TGA results indicate that the incorporation of MCNTs can improve the char residues significantly at high temperature. Based on the Raman spectra and TGA results, it can be concluded that the MCNTs could only catalyze the formation of more glassy carbon as the composites thermally decompose.

## Conclusion

In the present work, CNTs was modified by organic/inorganic silicon based compound through sol-gel process, PS/CNTs and PS/MCNTs composites with different particles contents have been prepared successfully through *in situ* polymerization. The TEM results indicate that the MCNTs disperse well in the PS matrix due to the improved interface interaction between PS and MCNTs. The incorporation of MCNTs into the PS matrix can improve the thermal and flame retardant properties of PS. The homodispersion of MCNTs in the PS matrix and the flame retardant element on the surface of MCNTs are the two main factors for the improved thermal stability and flame retardants properties. The PS/MCNTs composites form a stable silicon and phosphorus based char layers. It’s believed that the stable char layers can not only serve as an efficient insulating barrier to reduce the exposure of PS matrix to heat but also retard the releasing of the flammable gases.

## Data Availability

The raw data supporting the conclusions of this article will be made available by the authors, without undue reservation.
